# Changes in sleep phase and body weight of mobile health App users during COVID-19 mild lockdown in Japan

**DOI:** 10.1038/s41366-021-00890-7

**Published:** 2021-07-03

**Authors:** Yu Tahara, Takae Shinto, Kosuke Inoue, Farnaz Roshanmehr, Akito Ito, Mikiko Michie, Shigenobu Shibata

**Affiliations:** 1 Laboratory of Physiology and Pharmacology, School of Advanced Science and Engineering, Waseda University, Shinjuku-ku, Tokyo Japan; 2 Department of Epidemiology, Fielding School of Public Health, University of California, Los Angeles (UCLA), Los Angeles, CA USA; 3 Department of Social Epidemiology, Graduate School of Medicine, Kyoto University, Kyoto, Japan; 4 Asken Inc., Shinjuku-ku, Tokyo Japan

**Keywords:** Risk factors, Epidemiology, Weight management

## Abstract

**Objective and method:**

The stay-at-home order during the COVID-19 pandemic has restricted individuals’ social behaviors, and therefore, effected their lifestyle including sleep, diet, and physical activity. Using the cross-sectional study design with a large sample size (*N* = 30,275) from the mobile health App users in Japan, we show age-dependent lifestyle changes during a nonpunitive “mild lockdown” (from April to May 2020).

**Results:**

Sleep onset and offset were delayed on work-days but not on free-days with increased sleep duration and decreased social jetlag, and the changes were more evident in the younger population. Although average weight change was close to none because of the users’ characteristic (95% of App users try to lose weight), we investigated an association between lifestyle change and body-weight change. Participants who reported advanced sleep phase during mild lockdown described a weight decrease. In contrast, the delayed sleep phase reported a weight gain. The results were significant after adjustment of confounding factors including physical activity and meal changes.

**Conclusions:**

Although there is cumulative evidence showing a relationship between late chronotype and obesity, it is still unclear about the potential benefit of the chronotype management to control body weight. Thus, to the best of our knowledge, this is the first study investigating the association between chronotype and weight changes by leveraging a large cohort.

## Introduction

The potential impact of lifestyle change on health outcomes during the COVID-19 pandemic has received substantial attention. The stay-at-home order has restricted individuals’ social behaviors, and therefore, effected their lifestyle including sleep, diet, and physical activity. Since these lifestyles are strongly associated with obesity, which is one of the major public health issues worldwide [1], the current pandemic-related social restrictions increased the incidence of obesity [2–4]. In Japan, a nonpunitive “mild lockdown” under a declared state of emergency for COVID-19 was held April to May 2020 [5]. All residents were supposed to stay at home (not mandatory). All schools, entertainment places, shops, dining places, and event spaces were supposed to be closed, but essential services (transportation, hospital, grocery stores) were continued.

The aim of this study is to find the effect of COVID-19-related social restrictions on the mobile health App “Asken” users, who generally intend to lose weight. Asken is a food-log and food-coaching App and was downloaded about 5500,000 times in Japan. The App gives feedback of food contents based on the Dietary Reference Intakes for Japanese decided by the Ministry of Health, Labor, and Welfare. By cross-sectional large data (*N* = 30,275) from an online survey in this App, we show age-dependent sleep changes by this mild lockdown. Moreover, we are the first to describe an association between sleep phase shift and body-weight change during the pandemic.

## Results

**Fig. 1:**
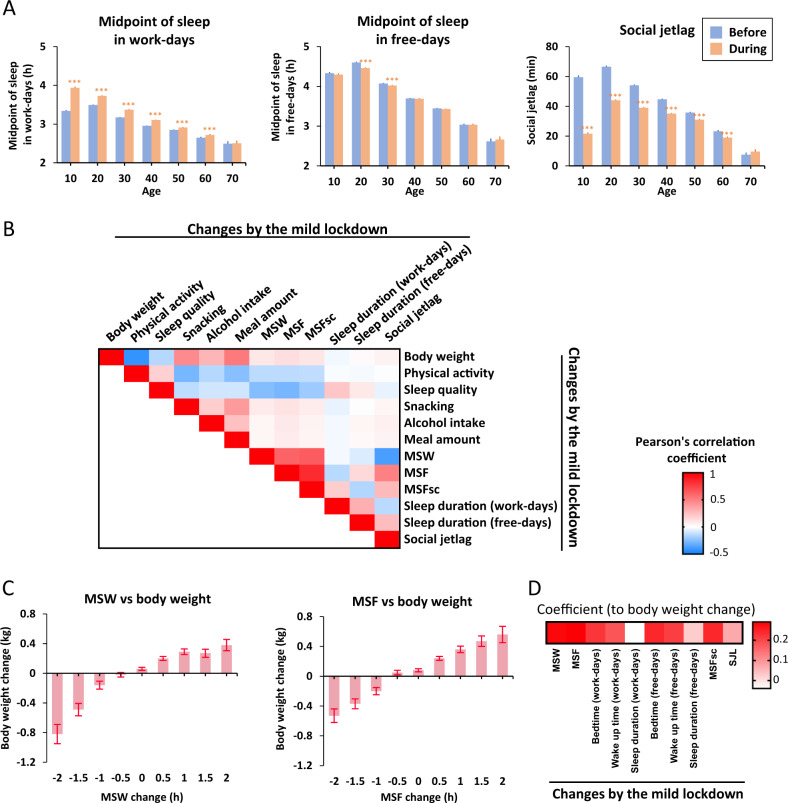
Effects of mild lockdown on the sleep and body weight. **A** Age-dependent sleep changes by mild lockdown. MSW midpoint of sleep in work-days, MSF midpoint of sleep in free-days, SJL social jetlag. Significant interaction effects (age × mild lockdown) by two-way repeated measure ANOVA were seen in each graph (*p* < 0.001). ***p < 0.001 between before and during the mild lockdown by post hoc Bonfferoni analysis. **B** Heatmap of Pearson’s correlation coefficient in each variable. **C** Correlation between the changes in MSW/MSF and body weight by the mild lockdown. Each bar shows the mean value (±standard error) of body-weight change at each category. The range of hours was restricted to −2 to 2 h because there are too few data points outside of this range (i.e., <−2 and >2 h). **D** Heatmap of coefficient value in the regression analysis to explain the body-weight change adjusting for age and sex. *N* = 30,275.

From 40,513 answers, a total of 30,275 subjects (age 10–70s; average 36.3 years old; male, *n* = 7949; female, *n* = 22,326) excluding shift workers and subjects who answered outliers were retrieved (Fig. S1 and Table S1). Participants include 73.7% of females due to the sex difference of the original user population in this App (Table S1 and Fig. S5). Data include normal distribution of chronotype (individual morningness and eveningness preferences), which is calculated from the mid-time of the sleep phase on free-days adjusted with sleep debt on work-days (MSFsc), and age-dependent phase-advancement of MSFsc as previously reported [6] (Figs. S2 and S3). We found that sleep onset, offset, and mid-time of the sleep phase were delayed on work-days but not on free-days by mild lockdown, and the changes were more evident in the younger population independent of sex (Figs. 1A, S2, S3, and S5). Sleep duration was also increased only on work-days (Figs. S2 and S3), which is correlated with the improvement of sleep quality (Fig. 1B; Pearson’s *r* = 0.222). Consistent with these findings, the social jetlag (weekly jetlag appeared by the sleep phase difference between work-days and free-days) was decreased especially in younger ages (Fig. 1A), but the change was not correlated with the sleep quality change (Fig. 1B; Pearson’s *r* = −0.048). Taken together, mild lockdown is associated with phase-delay and increased sleep duration in work-days, and decreased social jetlag.

Then, we analyzed body-weight change during the mild lockdown to understand the relation with a lifestyle change. Averaged weight change was 0.08 kg in this cohort (Table S1); it might be because of this App users’ characteristic. Physical activity, sleep quality, lifestyle regularity, snacking, meal amount, and alcohol by the mild lockdown were correlated with the change of body weight (Figs. 1B, S6, and S7). In addition, we found a clear correlation between the sleep phase (bedtime and wake up time on both work-days and free-days, MSW, MSF, or MSFsc) and body-weight change (Fig. 1B–D and S4). Participants who reported advanced sleep phases on both work-days and free-days during mild lockdown described a weight decrease. In contrast, participants with delayed sleep phase reported a weight gain. Although sleep phase changes were correlated with the lifestyle changes such as physical activity, sleep quality, and food intake (Fig. 1B), the correlation between sleep phase and body weight was consistent even after adjusting for age, sex, and those lifestyle changes (Fig. 1D and Table 1). Interestingly, improvement of sleep quality was strongly correlated with the advancement of the MSW, MSF, and MSFsc (Pearson’s *r* = −0.257, −0.283, −204, respectively; Figs. 1B and S6A). However, the correlation between sleep duration or social jetlag changes, and body-weight change was weak or not significant (Figs. 1B, S4 and Table 1). Some of the subjects (*n* = 10,355) recorded height and body weight using this App. We confirmed BMI change correlated with MSW and MSF after adjusting the age, sex, and those lifestyle changes (*p* < 0.01). In summary, chronotype shift during the mild lockdown was associated with the body-weight change.

**Table 1:** Association between sleep parameters and body-weight change.

	Model1			Model2		
	*β*	95% CI	*p* value	*β*	95% CI	*p* value
MSW change (h)	0.273	0.243, 0.302	<0.001	0.075	0.052, 0.099	<0.001
MSF change (h)	0.295	0.267, 0.322	<0.001	0.088	0.066, 0.110	<0.001
Sleep duration change (work-days, h)	−0.044	−0.068, −0.02	<0.001	−0.019	−0.038, −0.001	0.043
Sleep duration change (free-days, h)	0.026	0.002, 0.05	0.036	0	−0.019, 0.019	0.998
Social jetlag change (h)	0.083	0.05, 0.115	<0.001	0.032	0.006, 0.057	0.016

Multivariable regression analyses adjusted by age and sex (model1) and plus physical activity, sleep quality, meal amount, and snacking changes (model2).

*CI* confidence interval.

## Discussion

Here we found the improvement of social jetlag and sleep duration by the mild lockdown in Japan. Previous reports of delayed sleep phase on work-days and decreased social jetlag during the COVID-19 pandemic in Argentina [7] and a worldwide national online survey [8] indicate that this lifestyle change might be universal. Several studies have demonstrated the improvement of adolescent sleep debt and social jetlag by delaying school start time (e.g., [9]). Other studies showed an association of sleep phase and chronotype with genetic SNPs [10] and with age-dependent environmental factors [6]. Our findings of age-dependent lifestyle change by the mild lockdown corroborate such previous literature indicating the potential influence of social behaviors inducing morningness lifestyle on work-days. Most participants (61.5%) answered that sleep quality did not change during the mild lockdown in the current study. In the UK, by COVID-19 lockdown, although more people answered worsened sleep correlating with increased anxiety, improved sleep was correlated with the length of time spent outside [11]. In our study, sleep quality positively correlated with sleep duration change on work-days but not on free-days, suggesting that the sleep debt and quality were recovered on work-days by the mild lockdown. In contrast, morningness shift on both work-days and free-days correlated with improvement of sleep quality, suggesting that the delay of sleep phase on work-days and the reduction of social jetlag did not improve sleep quality during this pandemic.

To the best of our knowledge, this is the first study investigating the association between chronotype management and body-weight change during the COVID-19 pandemic using a large cohort from an online survey database. In addition, we adjusted for several lifestyle-related factors in our model. Social jetlag often seen in late chronotype is related to obesity [6]. Late chronotype is associated with late eating time and breakfast skipping, resulting in higher postprandial blood glucose levels and a higher risk of obesity [12]. Several studies found the correlation between chronotype and losing weight in their intervention or post-bariatric surgery patients [12]. Evening chronotype with late lunch exhibited slower weight-loss progress independent of the calorie intake and dietary composition [13]. However, an intervention study of chronotype management on body-weight control has never been done. Thus, the current pandemic-related lifestyle change was a great opportunity to examine the effect of the sleep phase alteration on body weight. In animal experiments, feeding patterns (e.g., skipping breakfast, late-night eating, or large portion dinner with small breakfast) induce obesity by changing the individual’s biological clock and modification of expression of metabolic genes controlled by the circadian-rhythm [14]. However, there is no appropriate model for animals to study human chronotype. Current data showed a correlation between chronotype shift and lifestyle change, including physical activity and food intake. This is consistent with previous findings displaying morning chronotype shows higher physical activity and better eating pattern [15]. In this context, our data proposed the possible relationship between sleep phase pattern and body-weight control, and the long-term intervention of chronotype to body-weight maintenance in humans will be examined in the future.

The limitations of our study include misclassification due to the self-reports, unmeasured and uncontrolled confounding, unclear temporal ordering between variables, and lack of generalizability to other populations given the various pandemic situations and political decisions. In summary, our findings suggest the possible modifications in sleep pattern and its association with body-weight change among the Japanese population during the mild lockdown. As obesity or disrupted circadian clock by social jetlag or irregular lifestyle exacerbate immune function [16], further investigation with longitudinal follow-up would be urgently needed to validate our findings, identify underlying mechanisms, and consider the effective strategy against the worldwide global health crisis.

## Supplementary information

Supplemental information
