# Supplementary material for: Changes in sleep phase and body weight of mobile health App users during COVID-19 mild lockdown in Japan
Source: Int J Obes (Lond). 2021 Jul 3;45(10):2277–80. doi: 10.1038/s41366-021-00890-7 (PMC8254445; doi:10.1038/s41366-021-00890-7)
Supplement: Supplementary file 1 — Supplemental information [file 41366_2021_890_MOESM1_ESM.pdf]

Supplemental Information

Changes in sleep phase and body weight of mobile health App users during COVID-19 mild lockdown in Japan

Yu Tahara, Takae Shinto, Kosuke Inoue, Farnaz Roshanmehr, Akito Ito, Mikiko Michie, and Shigenobu Shibata

Supplemental figures and figure legends

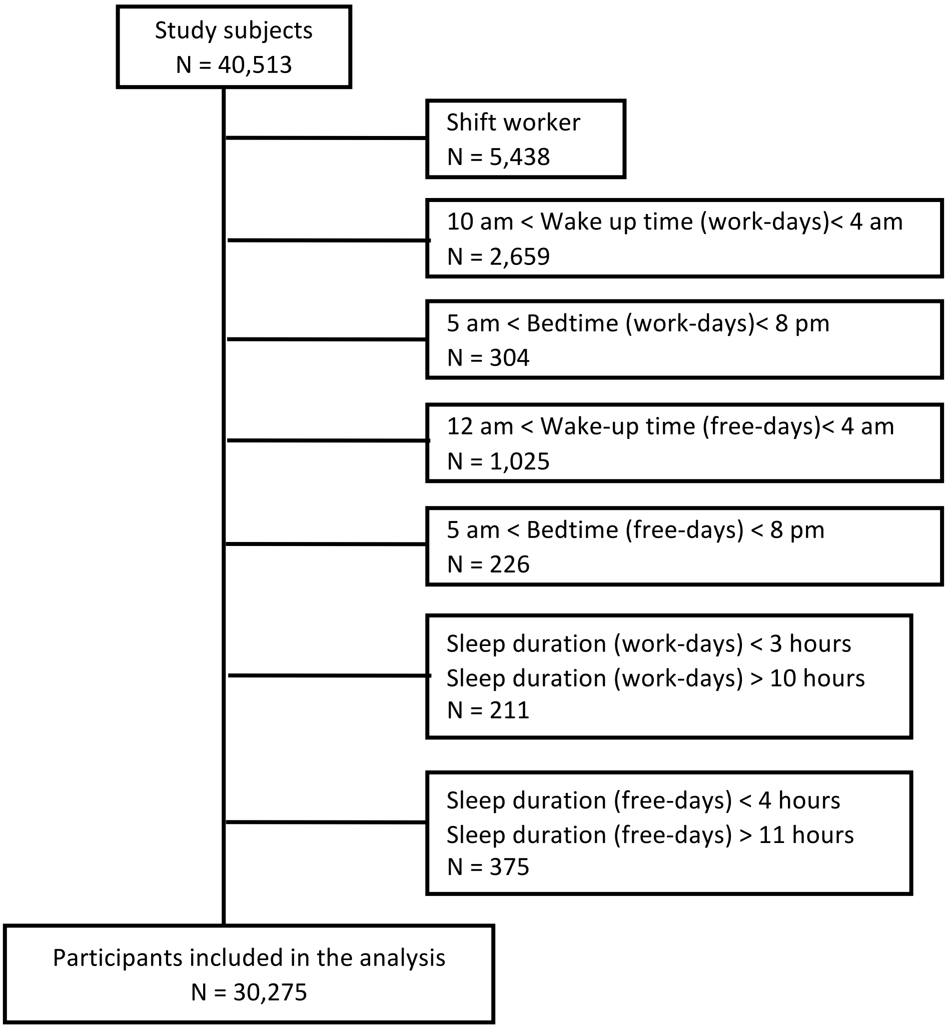

Figure S1. Study flow design. We excluded the subjects who answered as shift-workers at first, then we excluded the data with outliers of sleep phase or duration on work-days or free-days.

Table S1 Subject characterization and sex differences

	Male (n = 7,949)			Female (n = 22,326)			All (n = 30,275)		
	Before	During	Change	Before	During	Change	Before	During	Change
Body weight (kg)			-0.21			0.19			0.08
Physical activity (score)			2.57			2.38			2.43
Sleep quality (score)			2.98			2.91			2.93
Bedtime in work-days (h)	23.61	23.51	-0.10	23.74	23.82	0.08	23.71	23.74	0.03
Wake up time in work-days (h)	6.23	6.41	0.18	6.51	6.88	0.37	6.43	6.76	0.32
Bedtime in free-days (h)	23.82	23.75	-0.07	24.13	24.13	0.00	24.04	24.03	-0.02
Wake up time in free-days (h)	7.21	7.16	-0.05	7.86	7.78	-0.08	7.69	7.62	-0.07
Sleep duration in work-days (h)	6.62	6.90	0.28	6.76	7.06	0.30	6.73	7.02	0.29
Sleep duration in free-days (h)	7.39	7.41	0.02	7.74	7.66	-0.08	7.65	7.59	-0.05
MSW (h)	2.92	2.96	0.04	3.12	3.35	0.22	3.07	3.25	0.18
MSF (h)	3.51	3.46	-0.06	3.99	3.96	-0.04	3.87	3.82	-0.04
MSFsc (h)	3.07	3.15	0.07	3.46	3.60	0.14	3.36	3.48	0.12
Social jetlag (h)	0.59	0.49	-0.10	0.87	0.61	-0.26	0.80	0.58	-0.22

Score of physical activity and sleep quality was 5 choices from 1: worsened to 5: improved (see the details in method section).

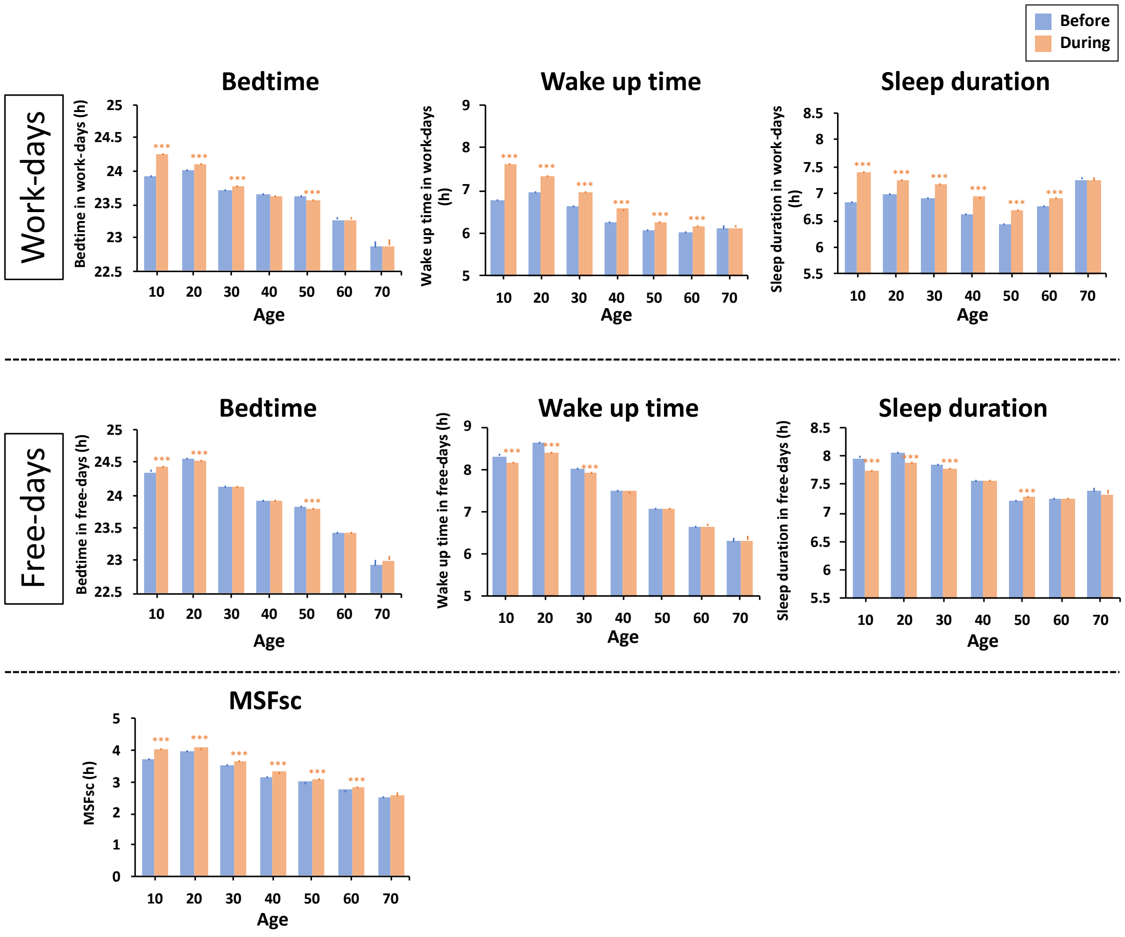

Figure S2 (related to Figure 1A). Age-dependent sleep changes (bedtime in work-days and free-days, wake up time in work-days and free-days, sleep duration in work-days and free-days, and MSFsc) by mild lockdown. Delayed bedtime and wake up time, and increased sleep duration between before and during the mild lockdown were seen in work-days but not in free-days. Significant interaction effects (age x mild lockdown) by two-way repeated measure ANOVA were seen in each graph ($p < 0.001$). ***$p < 0.001$ between before and during the mild lockdown by post-hoc Bonferroni analysis. Data are expressed as mean ± SEM.

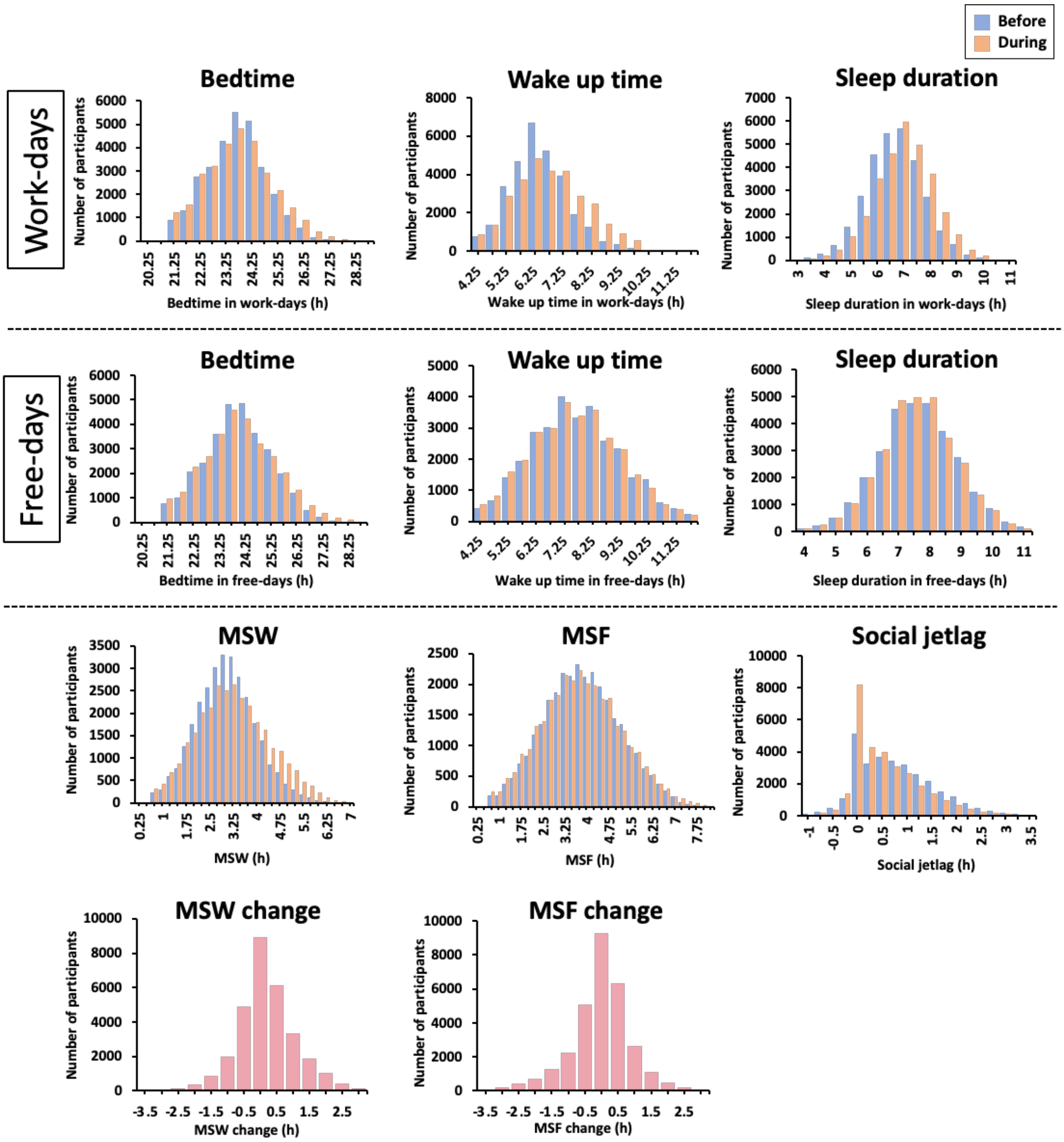

Figure S3 (related to Figure 1A). Participants distribution of each sleep parameter before and during the mild lockdown. The participants distribution also confirmed the phase-delay of sleep phase in the work-days and decreased social jetlag.

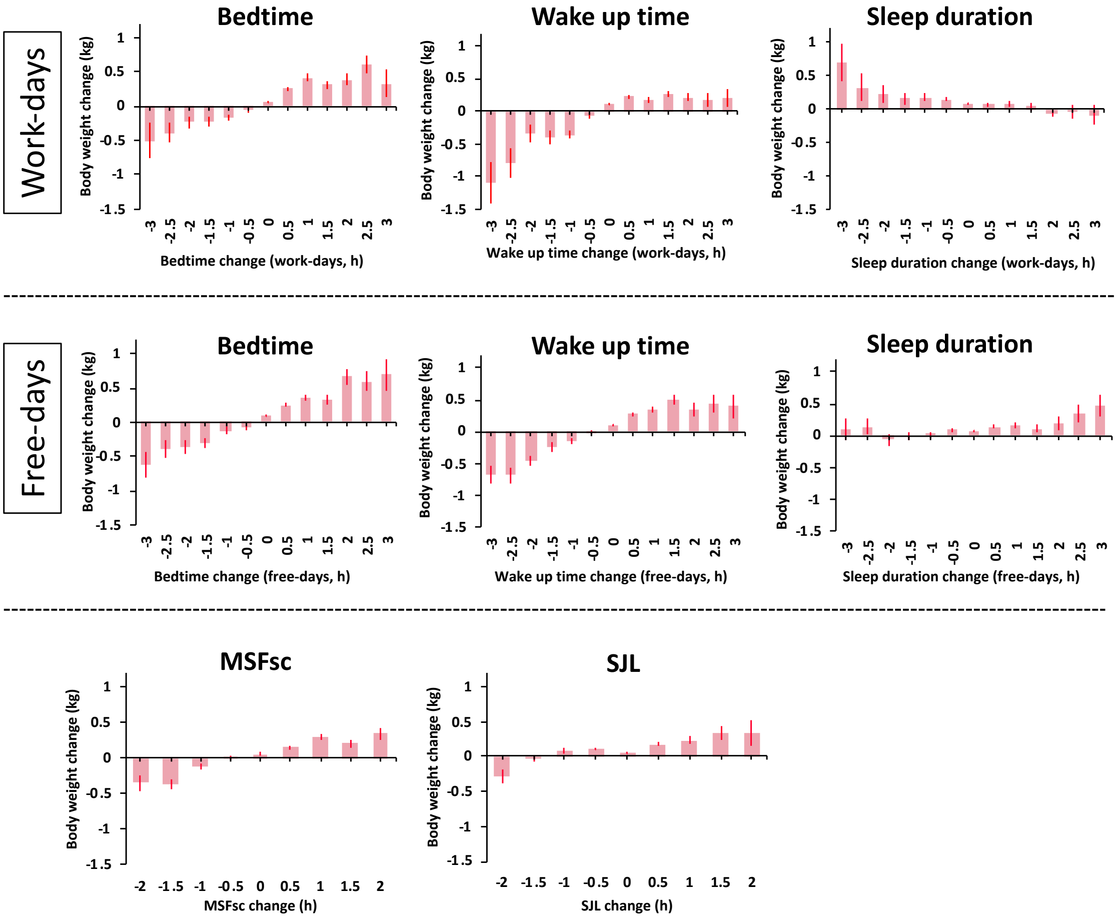

Figure S4 (related to Figure 1C). Correlation between the changes of each sleep parameter and body weight by mild lockdown. Clear correlation between sleep and body weight change was seen only in the bedtime and wake up time, but not in the sleep duration or social jetlag. Each bar shows the mean value (± standard error) of body weight change at each category. The range of hours was restricted because there are too few data points outside of this range (i.e., <2 hours and >2 hours).

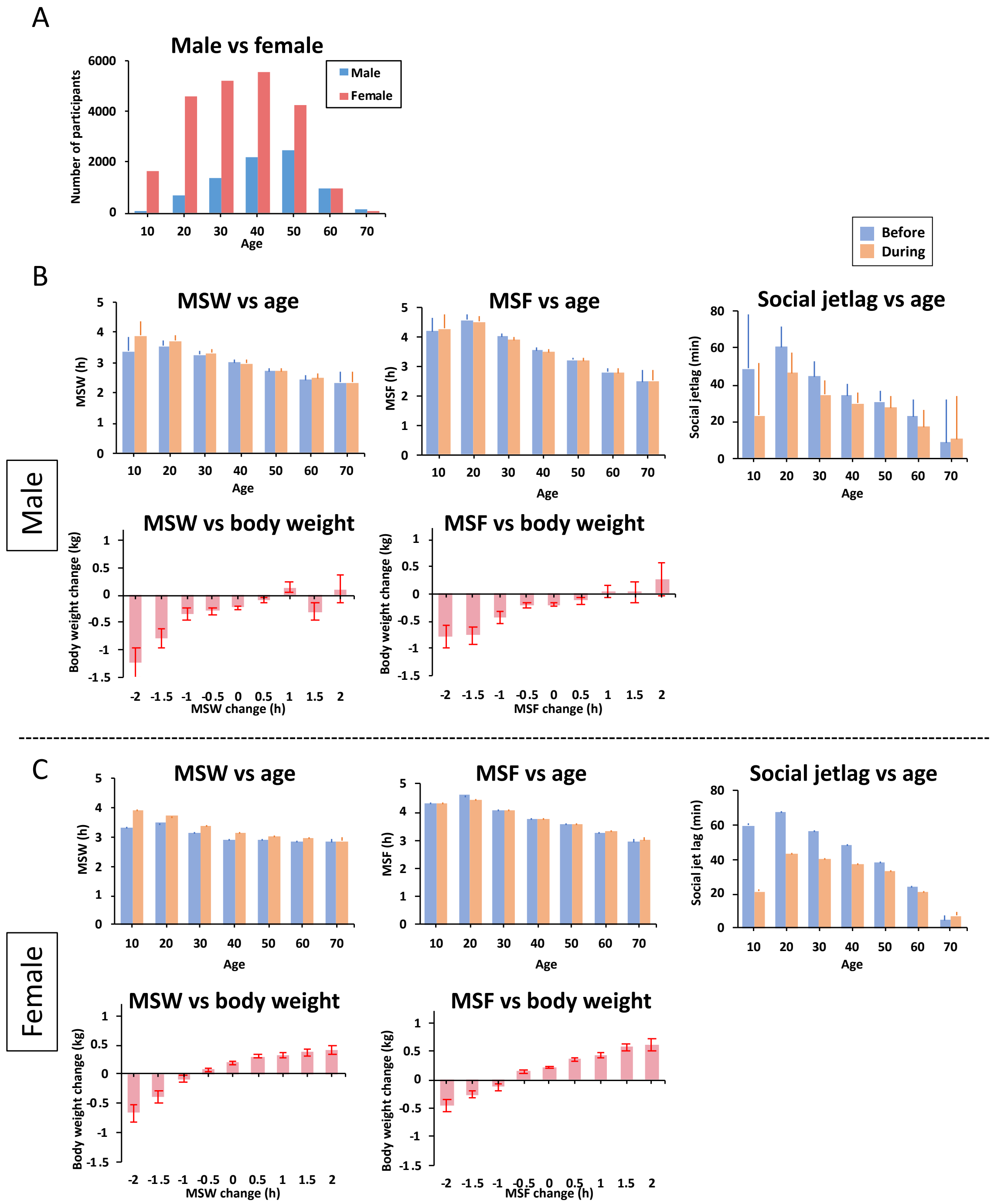

Figure S5 (related to Figure 1). Gender dependent analysis of the main findings. (A) Participants distribution of male and female in each age. (B, C) Sex difference of the main findings (B, males: n = 7,949; C, females: n = 22,326). Although population size is different in male and female, the main finding is the same in each gender.

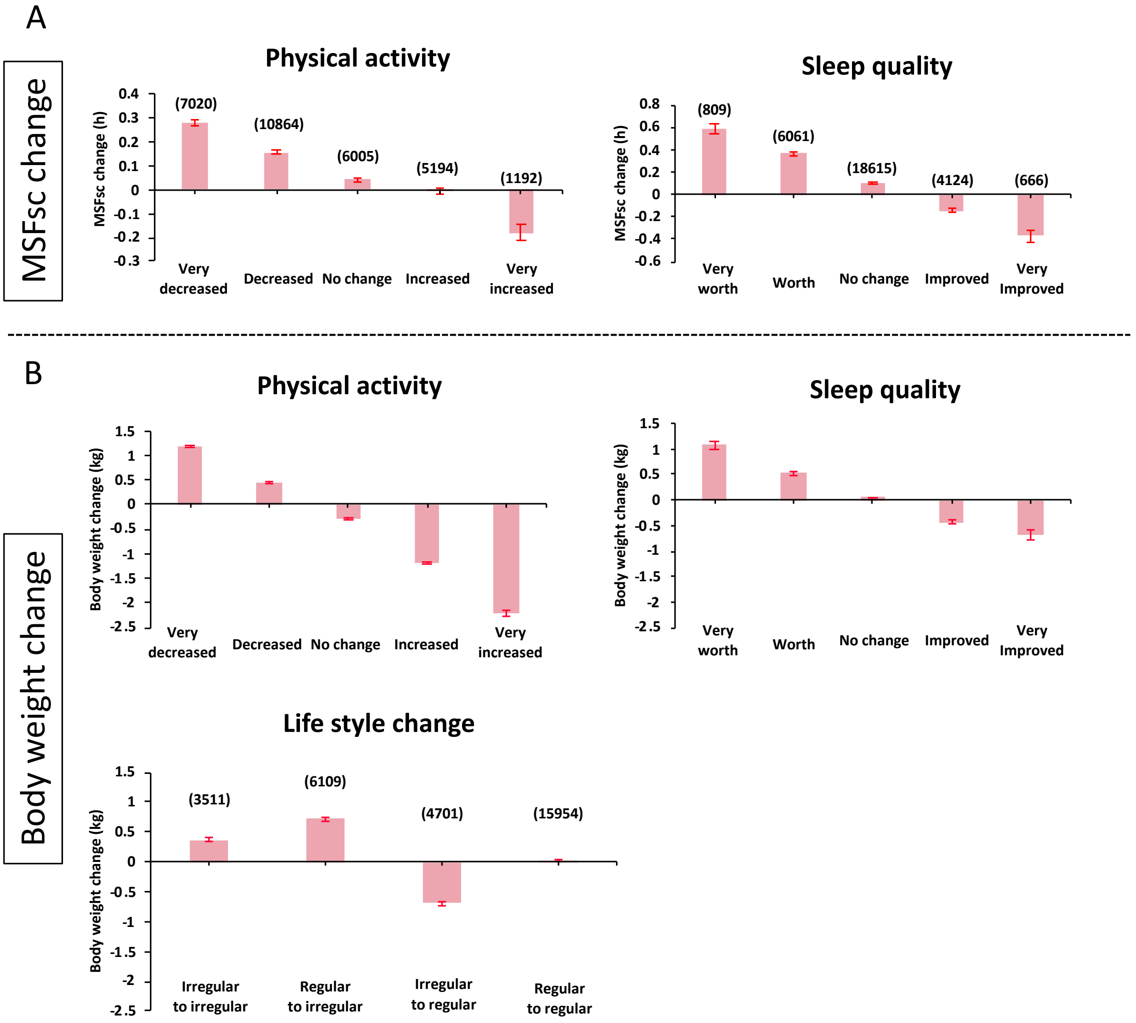

Figure S6. Correlation between the questionaries of physical activity, sleep quality, or life style, and MSFsc (A) or body weight (B) changes by mild lockdown. Numbers in the parenthesis above each bar indicates the number of participants. 59.1% of participants answered decreased physical activity, and 22.7% answered decreased sleep quality by the mild lockdown. In contrast, 21.1% increased physical activity and 15.8% improved sleep quality.

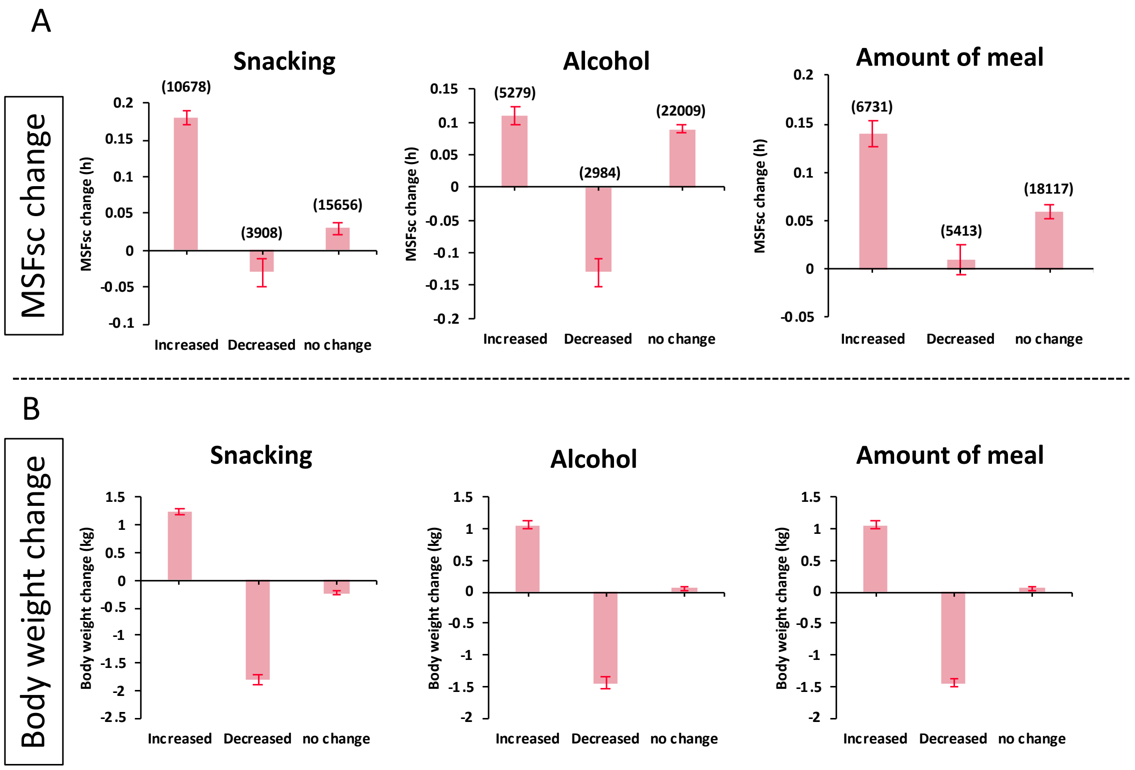

Figure S7 Changes in snacking, alcohol drinking, and the amount of meals are correlated with the changes in MSFsc (A) and body weight (B). Numbers in the parenthesis above each bar indicates the number of participants.

Methods

Data Collection

The mild lockdown associated with the COVID-19 pandemic by the government's announcement of "the statement of emergency" started on April 16th, 2020, and ended on May 14th, 2020, in Japan as a whole (but from April 7th to May 25th in some prefectures including Tokyo). All residents were supposed to stay at home (not mandatory). All schools, entertainment places, shops, dining places, and event spaces were supposed to be closed, but essential services (transportation, hospital, grocery stores) were continued.

To understand the mild lockdown effect, a cross-sectional online survey was conducted from May 25th, 2020 to June 1st, 2020, in Japan to the APP users voluntarily using a phone APP "Asken" (produced by asken Inc., Tokyo, Japan). "Asken" is a popular food-log and food-coaching APP, downloaded about 5,500,000 times (https://www.asken.inc/eng), always ranked in Top 3 for the health APP categories in Japan [1]. The self-reported food log accumulated in the APP is also reliable for the research [1]. Since most users (almost 95%) use this APP to reduce their body weight and females might be more in interested their body shape than males, 70% of users of this APP are female. When conducting the online survey with informed consent, we announced that 25 people would be given 500 Japanese yen in the lottery. The ethics review committee on research with human subjects in Waseda University approved this experiment (No. 2020-046), and the guidelines laid down in the Declaration of Helsinki was followed.

Participants

As shown in Figure S1, we initially excluded shift workers (13.4% of participants, 5,438 subjects) and those who answered meaningless data or outliers of the sleep phase and duration from normal distribution. The final analytical sample was 30,275 subjects out of 40,513 subjects were used for the current analysis. Data includes 73.7% of female and 10s-70s of age (Fig. S5).

Online survey contents

The questionnaire includes personal characteristics (sex, age, prefecture of residence, shift worker or not) and others are listed below.

Q1: Did you try to lose weight during this mild lockdown?

(1: Yes; 2: No)

Q2: Did your body weight change during this mild lockdown?

(1: Decreased; 2: No change; 3: Increased)

Q3: How much did your body weight change?

(1: 0.1-1.0 kg; 2: 1.1-2.0 kg; 3: 2.1-3.0 kg; 4: 3.1-4.0 kg; 5: Over 4 kg; 6: No change)

Q4: Did your physical activity or exercise amount change during this mild lockdown?

(1: Very decreased; 2: Decreased; 3: No change; 4: Increased; 5: Very increased)
Q5: How was your eating habit during this mild lockdown?
(1: Before 1-2 meals/day but 3 meals/day during quarantine; 2: Before 3 meals/day but 1-2 meals/day during quarantine; 3: No change)
Q6: How was your snacking habit during this mild lockdown?
(1: Increased snacking; 2: Decreased snacking; 3: No change)
Q7: How was your alcohol drinking during this mild lockdown?
(1: Increased; 2: Decrease; 3: No change)
Q8: How was your total eating amount during this mild lockdown?
(1: Increased; 2: Decrease; 3: No change)
Q9-16: Timing of sleep phase (go to bed and wake up) in work-days and free-days, before and during mild lockdown?
(Participants choose time windows every 30 min.)
Q17: How was your lifestyle changed by this mild lockdown?
(1: Irregular sleep cycle before and during quarantine; 2: Regular sleep cycle before, but became irregular during quarantine; 3: Irregular sleep cycle before, but became regular during quarantine; 4: Regular sleep cycle before and during quarantine)
Q18: How was your sleep quality changed by this mild lockdown?
(1: Very worth; 2: worth, 3: No change, 4: improved, 5: Very improved)

From Q9-16, based on the references [2, 3], we calculated sleep duration, middle time of sleep phase in work-days (MSW) and free-days (MSF), adjusted MSF (MSFsc = MSF- ((sleep duration in free-days) - (sleep duration in work-days))/2), and social jetlag (= MSF-MSW). Some of the subjects (n = 10,363) recorded height and body weight using this APP. Since actual body weight was also recorded in some subjects at the last week before starting the mild lockdown (Apr 7th-14th) and the last week of mild lockdown (May 7th - 14th) (n = 10,355), we confirmed that the body weight change collected by an online survey is correlated with that recorded in APP (Pearson's r = 0.607).

Statistical Analysis

Sample size was calculated by the power analysis with our previous survey data. Data are expressed as the mean ± SEM. $P < 0.05$ was considered to indicate statistical significance. Equal variance and normal distribution tests were performed to select the appropriate statistical approach. Parametric analyses were conducted using a one-way, two-way, or two-way repeated measures ANOVA with Tukey, Dunnett, or Student's t-tests for post-hoc analysis. The SPSS Statistics version 26 (IBM, NY, USA), Stata version 15 (StataCorp, TX, USA) were used for the

statistical analysis.

Additional references

1. Shimpo, M., Fukkoshi, Y., and Akamatsu, R. (2014). Correlations between self-efficacy in resisting six temptations and dietary energy and macronutrient intake at each meal. Eat Behav *15*, 563-566.
2. Roenneberg, T., Wirz-Justice, A., and Merrow, M. (2003). Life between clocks: daily temporal patterns of human chronotypes. Journal of biological rhythms *18*, 80-90.
3. Kitamura, S., Hida, A., Aritake, S., Higuchi, S., Enomoto, M., Kato, M., Vetter, C., Roenneberg, T., and Mishima, K. (2014). Validity of the Japanese version of the Munich ChronoType Questionnaire. Chronobiology international *31*, 845-850.
